# Evaluation of alveolar recruitment maneuver on respiratory resistance during general anesthesia: a prospective observational study

**DOI:** 10.1186/s12871-020-01182-9

**Published:** 2020-10-17

**Authors:** Junko Nakahira, Shoko Nakano, Toshiaki Minami

**Affiliations:** Department of Anesthesiology, Osaka Medical College, 2-7 Daigaku-machi, Takatsuki, Osaka, 569-8686 Japan

**Keywords:** Respiratory resistance, Alveolar recruitment maneuver, Forced oscillation technique

## Abstract

**Background:**

Alveolar recruitment maneuvers enable easily reopening nonaerated lung regions via a transient elevation in transpulmonary pressure. To evaluate the effect of these maneuvers on respiratory resistance, we used an oscillatory technique during mechanical ventilation. This study was conducted to assess the effect of the alveolar recruitment maneuvers on respiratory resistance under routine anesthesia. We hypothesized that respiratory resistance at 5 Hz (R5) after the maneuver would be decreased after the lung aeration.

**Methods:**

After receiving the ethics committee’s approval, we enrolled 33 patients who were classified with an American Society of Anesthesiologists physical status of 1, 2 or 3 and were undergoing general anesthesia for transurethral resection of a bladder tumor within a 12-month period from 2017 to 2018. The recruitment maneuver was performed 30 min after endotracheal intubation. The maneuver consisted of sustained manual inflation of the anesthesia reservoir bag to a peak inspiratory pressure of 40 cmH_2_O for 15 s, including 5 s of gradually increasing the peak inspiratory pressure. Respiratory resistance was measured using the forced oscillation technique before and after the maneuver, and the mean R5 was calculated during the expiratory phase. The respiratory resistance and ventilator parameter results were analyzed using paired Student’s *t-*tests, and *p* < 0.05 was considered statistically significant.

**Results:**

We analyzed 31 patients (25 men and 6 women). R5 was 7.3 ± 1.6 cmH_2_O/L/sec before the recruitment maneuver during mechanical ventilation and was significantly decreased to 6.4 ± 1.7 cmH_2_O/L/sec after the maneuver. Peak inspiratory pressure and plateau pressure were significantly decreased, and pulmonary compliance was increased, although the values were not clinically relevant.

**Conclusion:**

The recruitment maneuver decreased respiratory resistance and increased lung compliance during mechanical ventilation.

**Trial registration:**

Name of registry: Japan Medical Association Center for Clinical Trials.

Trial registration number: reference JMA-IIA00136.

Date of registration: 2 September 2013.

URL of trial registry record: https://dbcentre3.jmacct.med.or.jp/JMACTR/App/JMACTRE02_04/JMACTRE02_04.aspx?kbn=3&seqno=3582

## Background

Lung-protective ventilation using a low tidal volume is the standard of care for mechanically ventilated patients with acute respiratory distress syndrome [1] and was recently demonstrated to significantly improve postoperative outcomes in patients undergoing surgery [2–5]. Alveolar recruitment maneuvers (ARMs), which are used to reopen collapsed lungs, and positive end-expiratory pressure (PEEP) are lung-protective ventilation strategies [6–8]. Using recruitment maneuvers to open the lungs improves the effectiveness of PEEP for gas exchange during mechanical ventilation [9–11]. Although many studies have reported the effectiveness of low tidal volume ventilation and ARMs [2–4, 12–15], no studies have evaluated ARMs independently of the PEEP level [16]. The definition of ARMs varies among studies. An automatic ARM is an automated stepwise recruitment maneuver with PEEP-titration [17]. Although automatic ARMs are widely used, most hospitals use ventilators without an automatic ARM mode. We routinely use a sustained inflation ARM, which transiently applies a high-pressure static increase in airway pressure for 10–15 s.

The forced oscillation technique (FOT) is used to measure respiratory impedance by measuring the relationship between pressure waves applied externally to the respiratory system and the resulting respiratory airflow. The FOT device measures respiratory resistance at 5 Hz (R5), which includes the resistance in the oropharynx, larynx, trachea, bronchi, pulmonary alveolus, and chest wall tissue. Exertional breathing maneuvers are not required during FOT measurement. MostGraph-01® (Chest MI, Tokyo, Japan) is a noninvasive device that measures respiratory resistance using broadband frequency FOT. We previously reported using this device to measure increased respiratory resistance after general anesthesia [18–21].

Because few studies have evaluated ARMs independently of the PEEP level, we investigated the effect of ARMs on respiratory status during surgery. We hypothesized that compared with respiratory compliance, tidal volume and partial pressure of oxygen in the arterial blood (PaO_2_), respiratory resistance would be a better parameter for detecting the effects of ARMs in patients with mostly intact respiratory systems. This study was conducted to assess the effect of ARMs on respiratory resistance during routine anesthesia. We hypothesized that the R5 would be decreased after the ARM because of the lung aeration and that the tidal volume would be increased, even under volume-controlled ventilation.

## Methods

The ethics committee of Osaka Medical College, Japan (approval reference number 1252) approved the study, which was conducted in accordance with the declaration of Helsinki (1964). All participants provided written informed consent. The study was registered at the Japan Medical Association Center for Clinical Trials (reference JMA-IIA00136). We enrolled 33 patients who were classified as American Society of Anesthesiologists physical status 1, 2 or 3 and undergoing general anesthesia for transurethral resection of a bladder tumor within a 12-month period from 2017 to 2018. Exclusion criteria included substantive abnormalities in spirometry (forced expiratory volume in 1 s < 50% of predicted volume; forced vital capacity < 50% of predicted volume), active asthma (requiring bronchodilator therapy or coughing or wheezing at rest), preoperative fractional nitric oxide concentration in exhaled breath (FeNO) > 50 ppb [22], previous lung surgery, history of chronic obstructive pulmonary disease requiring bronchodilator therapy, home oxygen therapy or having had a respiratory tract infection within the previous 3 months. The primary outcome was the difference between the pre-ARM R5 and post-ARM R5 during ventilation. The secondary outcome was the difference in tidal volume and lung compliance.

### Preoperative measurements

Respiratory examinations were performed, including spirometry without bronchodilation and FeNO measurement. Spirometry was performed within a month before surgery using a spirometer (System 21 device®, Minato Igkagaku, Osaka, Japan) and FeNO was measured using NIOX VERO® (Aerocrine, Aolna, Sweden) the day before surgery. Respiratory resistance was measured using the MostGraph-01® the day before and the day after surgery. Patients sat with a nose clip and their cheeks supported firmly during the respiratory resistance measurements.

### Anesthetic management

A standardized anesthetic technique was used. Anesthesia was induced with 1.0–1.5 mg/kg intravenous propofol, 0.7–0.9 mg/kg rocuronium, a continuous infusion of 0.4 μg/kg/min remifentanil and 2.0–3.0% inhaled sevoflurane. The trachea was intubated with a tube that had an internal diameter of 7.0 mm for women and 8.0 mm for men (Portex Soft Seal®, Smiths Medical, Kent, UK). Anesthesia was maintained with inhaled sevoflurane, intravenous remifentanil and intravenous rocuronium at 4–6 μg/kg/min. The sevoflurane concentration was titrated over 1.5% at the discretion of the attending anesthetist according to a bispectral index monitor that was controlled between 40 and 60. At the end of the surgery, patients were administered 1000 mg intravenous acetaminophen for pain relief, followed by 1.5–2.0 mg/kg (maximum 200 mg) intravenous sugammadex. Tracheal suctioning was performed once or twice before patients were extubated. The endotracheal tube was removed when patients could communicate and breathe spontaneously with sufficient tidal volume. Supplementary oxygen at 6 L/min was administered via facemask immediately after extubation.

### Devices and measurement procedures during anesthesia

After inducing anesthesia, all patients were mechanically ventilated using the volume-controlled mode with an inspiratory/expiratory ratio of 1:2, an inspiratory pause time/total inspiratory time ratio of 0.1, tidal volume of 8 mL/kg ideal body weight with a PEEP of 5 cmH_2_O, a respiratory frequency of 10–12 breaths/min to maintain the end-tidal carbon dioxide from 35 to 45 mmHg, and an oxygen inspiratory fraction of 0.4 (Drägel Fabius®, Dragel Medical, Lubeck, Germany). The ARM was performed 30 min after tracheal intubation and consisted of sustained manual inflation of the anesthesia reservoir bag to a peak inspiratory pressure of 40 cmH_2_O for 15 s, including gradually increasing the peak inspiratory pressure for 5 s. Before the ARM, the peak airway pressure, plateau pressure, PEEP, and tidal volume were obtained from the ventilation monitor. Dynamic respiratory compliance (Cdyn) and static pulmonary compliance (Cst) were calculated as Cdyn = tidal volume/(peak pressure − PEEP) and Cst = tidal volume/(plateau − PEEP).

**Fig. 1:**
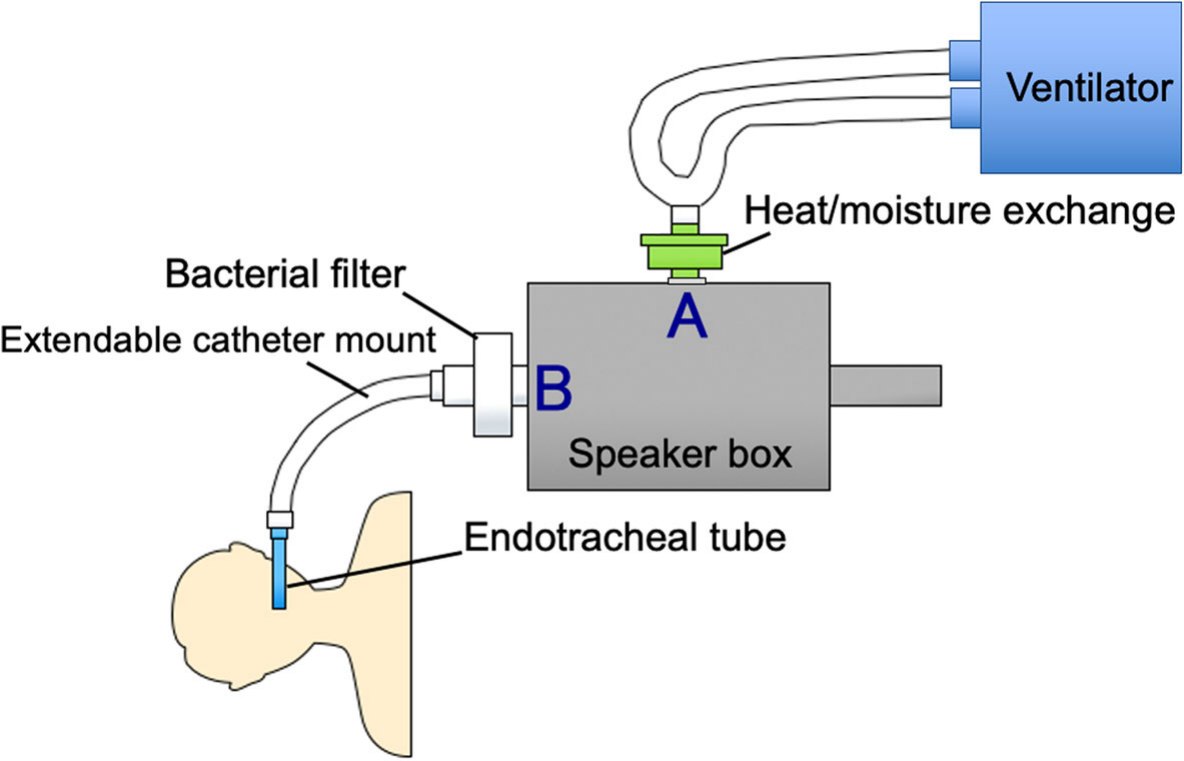
Schema of respiratory resistance measurements using the MostGraph-01® during mechanical ventilation. A is the connection between the speaker box and ventilator circuit. B is the connection between the speaker box and examinee. The speaker box is composed of a loud speaker and a pressure and flow sensor

**Fig. 2:**
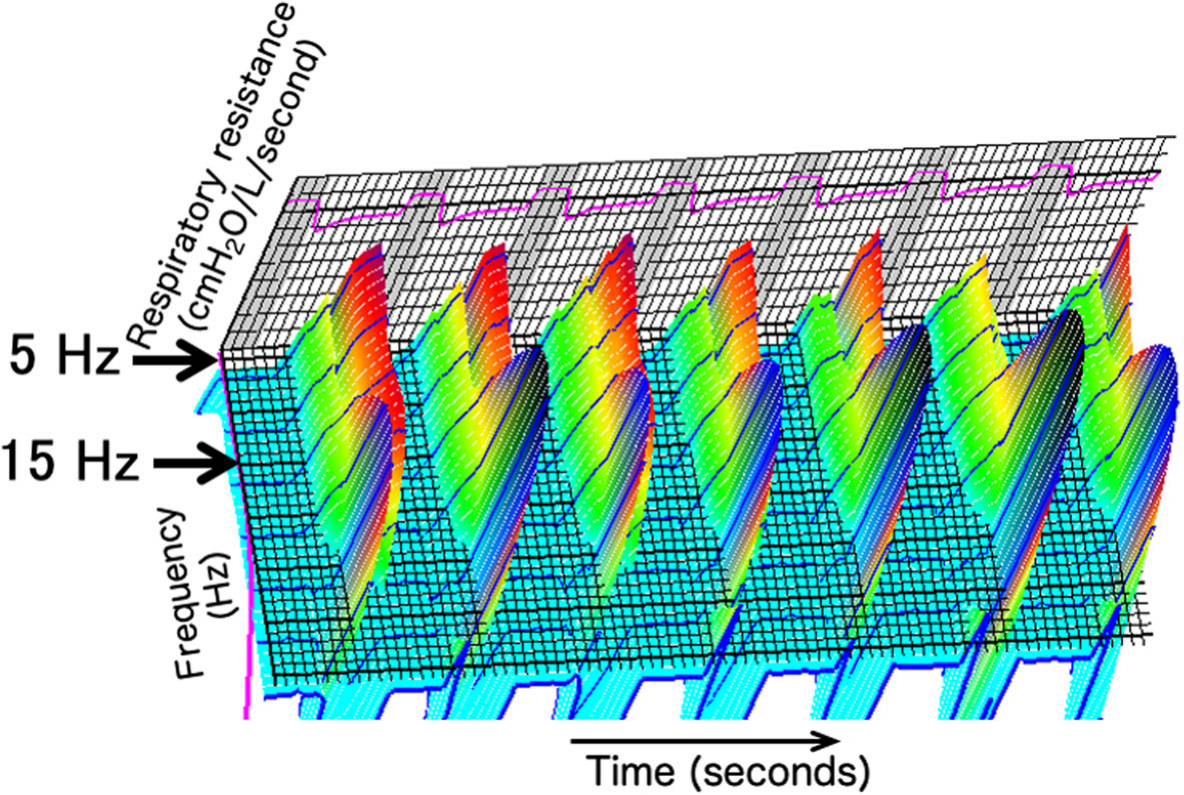
Example of respiratory resistance during mechanical ventilation. Three-dimensional graph of respiratory resistance with a frequency range from 4 to 35 Hz is shown. High respiratory resistance over 15 Hz indicates fluttering of a check valve in the ventilator

**Fig. 3:**
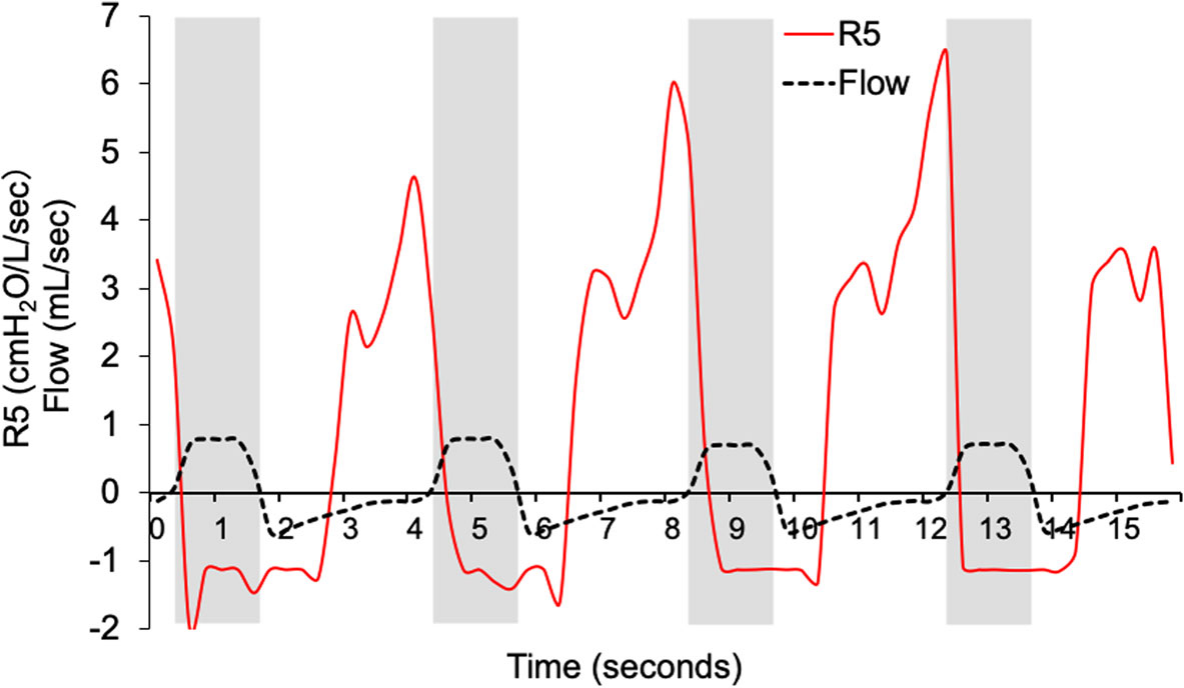
Example of respiratory resistance at 5 Hz during mechanical ventilation. Gray zones represent the inspiratory phase. The mean respiratory resistance at 5 Hz was calculated after baseline adjustment using the mean value of the latter half of the inspiratory phase

We modified this device to measure the R5 during ventilation using an endotracheal tube under general anesthesia. We used the MostGraph-01®, which was modified to measure respiratory resistance during mechanical ventilation (Figs. 1, 2) as previously described [23]. We put a connection on the vent to connect a loud speaker to the ventilator and endotracheal tube. The company that made and sold this device (Chest MI) made this modification after giving us their permission. Thirty minutes after inducing anesthesia, we measured the respiratory resistance using the modified device. During this measurement, the ventilator setting was changed to a tidal volume of 16 mL/kg ideal body weight with zero PEEP and an oxygen inspiratory fraction of 0.6. We measured the respiratory resistance again using the MostGraph-01® immediately after the ARM, changed the ventilator setting back to the previous setting and measured the peak airway pressure, plateau pressure, PEEP, and tidal volume again. Offline data from the MostGraph-01® were analyzed after each operation (Fig. 3). The mean respiratory resistance at 5 Hz was calculated after baseline adjustment using the mean value of the latter half of the inspiratory phase.

### Statistical analysis

Our preliminary measurements for the pre-ARM R5 and post-ARM R5 were 5.49 ± 1.25 cm H_2_O/L/s (mean ± SD) and 4.88 ± 1.35 cm H_2_O/L/s, respectively; therefore, the standard deviation was considered to be 1.25, and the expected difference in R5 was considered to be 1.25 cm H_2_O/L/sec. The sample size was determined from our preliminary R5 study of 8 patients using a subglottic airway device. A sample size of 33 patients was required to obtain 80% power between the pre-ARM and post-ARM at an α error level of 5% and an intragroup difference of 1.25 cm H_2_O/L/s in R5. Data are expressed as the mean ± SD or median (interquartile range, 25–75%) according to the variable distribution. Normality was analyzed using the Shapiro-Wilk test. The MostGraph-01® and ventilator parameter results were analyzed using paired Student’s *t*-tests; *p* < 0.05 was considered statistically significant. All statistical analyses were performed using GraphPad Prism 6 software (GraphPad Software, La Jolla, CA, USA).

## Results

For two of the 33 patients, the respiratory resistance measurement during mechanical ventilation was saved incorrectly; therefore, 31 patients (25 men and 6 women) were included in the final analysis. No patients experienced any serious perioperative events. No patient showed a preoperative FeNO > 50 ppb. No patient had < 96% oxygen saturation as measured by pulse oximetry. Table 1 shows the patients’ characteristics. The preoperative R5 was 2.6 ± 0.9 cmH_2_O/L/sec; the postoperative R5 was 2.8 ± 0.9 cmH_2_O/L/sec.

**Table 1:** Patient characteristics and operative results (*n*=31)

Characteristics	
Male/female (n)	25/6
Age (years)	69 (63-78)
Height (cm)	164.7±7.7
Body weight (kg)	65.8±10.9
Ideal body weight (kg)	59.8±5.5
Body mass index	24.2±3.0
Body surface area (m^2^)	1.72±0.17
ASA physical status I/II/III (n)	1/23/7
Comorbidities (n)	
Hypertension	10
Diabetes	6
Dyslipidemia	3
Atrial fibrillation	2
Dialysis	1
Coronary artery disease	1
Ex-/current smoker (n)	15
Brinkman index	300 (0-700)
VC (L)	3.4±0.8
%VC (% predicted)	97.5±11.6
FVC (L)	3.3±0.8
%FVC (% predicted)	98.4±12.1
FEV1.0 (L)	2.5±0.6
%FEV1.0 (% predicted)	76.2 (71.6-79.8)
FEV1.0/FVC (%)	73.0±11.0
FeNO (ppb)^a^	17 (15-21)
Preoperative R5 (cmH_2_O/L/second)	2.6±0.9
Postoperative R5 (cmH_2_O/L/second)	2.8±0.9
Operation time (minutes)	31 (21-55)
Anesthesia time (minutes)	79 (65-93)
Fluid infusion (mL)	400 (250-550)
Internal diameter of tracheal tube 7/7.5/8 mm (n)	6/1/24

Values are the mean±SD or median (percentile, 25%–75%) or number (n).

Brinkman index, defined as the number of cigarettes smoked per day multiplied by smoking years, was calculated only for smokers and ex-smokers.

*ASA* American Society of Anesthesiologists, *VC* Vital capacity, *FVC* Forced vital capacity, *FEV1.0* Forced expiratory volume in the first second.

^a^Data were detected in 17 cases.

R5 was significantly decreased after the post-ARM during mechanical ventilation (Table 2). The R5 was 7.3 ± 1.6 cmH2O/L/sec during mechanical ventilation before the recruitment maneuver; the R5 was significantly decreased to 6.4 ± 1.7 cmH_2_O/L/sec after the maneuver. Peak inspiratory airway pressure and plateau pressure were significantly decreased. Pre- and post-ARM lung compliance (Cdyn) were 47.0 ± 8.8 mL/cmH_2_O and 50.0 ± 8.9 mL/cmH_2_O, respectively. Pre- and post-ARM Cst were 58.3 ± 13.9 mL/cmH_2_O and 63.1 ± 13.3 mL/cmH_2_O, respectively. Lung compliance significantly increased after the ARM. Post-ARM tidal volume on the ventilator measurement was significantly increased compared with the pre-ARM tidal volume but not on the MostGraph measurement.

**Table 2:** Respiratory effects of the alveolar recruitment maneuver (*n*=31)

	Pre-ARM	Post-ARM	*P* value
MostGraph® measurement			
Tidal volume (mL)	447±55	458±53	0.077
R5 (cmH_2_O/L/second)	7.3±1.6	6.4±1.7	0.001
Ventilator measurement			
Tidal volume setting (mL)	497±53	497±53	NA
Actual tidal volume (mL)	480±56	488±56	0.026
Peak inspiratory pressure (cmH_2_O)	15.5±1.7	15.0±1.5	<0.001
Plateau pressure (cmH_2_O)	13.6±1.6	13.0±1.4	<0.001
PEEP (cmH_2_O)	5±0	5±0	NA
Cdyn (mL/cmH_2_O)	47.0±8.8	50.0±8.9	<0.001
Cst (mL/cmH_2_O)	58.3±13.9	63.1±13.3	<0.001

*ARM* Alveolar recruitment maneuver, *Cdyn* Dynamic respiratory compliance, *Cst* Static pulmonary compliance, *NA* Not applicable, *PEEP* Positive end-expiratory pressure.

## Discussion

This was the first study to evaluate the effect of ARMs on respiratory resistance. R5 represents respiratory resistance, including resistance in the oropharynx, larynx, trachea, bronchi, lungs, and chest wall tissue. This study showed that ARMS improved the R5 under general anesthesia, increased the lung compliance, and reduced the peak and plateau pressures. An increase in lung compliance immediately after applying the ARM might reflect a reduced atelectasis. A decrease in R5 suggests an improved Cdyn. Although the effect of ARMs on lung compliance is controversial [13], many studies have reported a statistically significant increase in lung compliance after ARMs in patients undergoing surgery [2, 12–15]. We hypothesized that respiratory resistance would be a better parameter for measuring the effects of ARMs in patients with intact respiratory systems; however, this hypothesis was incorrect.

A 2006 multicenter observational study in France reported that ARMs were only used in 7% of patients [24]; however, ARMs are widely used to improve arterial oxygenation [6]. Various ARMs have been proposed, including 1) sustained high-pressure inflation, 2) high tidal volumes and inspiratory pressures delivered intermittently, and 3) stepwise increases in PEEP and/or airway plateau inspiratory pressure [25]. However, the optimal method for ARMs remains uncertain. ARM use is not standardized and is left to the individual physician based on their experience. In this study, no automatic ARM was available for our ventilation machine; therefore, we performed sustained inflation as the ARM. Briefly, the manual ARM consisted of applying a continuous positive airway pressure of 30 cmH_2_O for 30 s [26]. A study reported that a plateau “opening pressure” of 40 cmH_2_O for 7–8 s effectively opened all alveoli in a nonobese patient with healthy lungs [27]. We used a continuous positive airway pressure of 40 cmH_2_O for 10 s in this study, primarily to prevent significant hemodynamic changes. ARM refers to a transient increase in transpulmonary pressure induced by increased intrathoracic pressure and decreased venous return, leading to a decrease in left ventricular end-diastolic areas and stroke volume [26, 28]. Importantly, automatic ARMs can cause hypotension [2, 13].

Contemporary anesthetic ventilators often include options to display the pressure-volume loop and calculated dynamic compliance values. Comparing the pre- and post-ARM lung compliance enables easier detection of the ARM’s effectiveness [26]. Automatic ARMs can be scheduled using the latest ventilation machine models. Previous studies performed the ARM as soon as mechanical ventilation was initiated, which was repeated after any discontinuation from the ventilator [13, 29]. Measuring respiratory resistance enables determining the best ARM for specific situations when patients are undergoing surgery.

Some studies have reported beneficial effects from ARMs. A randomized controlled trial revealed fewer incidences of postoperative nausea and vomiting after gynecological laparoscopic surgery [30]. Additionally, ARMs may reduce the incidence of shoulder pain following laparoscopic surgery [31, 32]. Our results showed no clinical significance regarding postoperative complications.

**Fig. 4:**
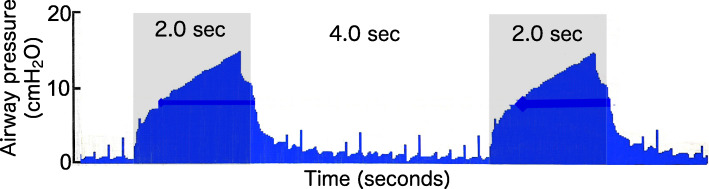
Airway pressure wave detected using a ventilator sensor during the respiratory resistance measurement. Airway pressure including oscillation pressure is shown. The ventilator settings were as follows: respiratory rate: 10/min, inspiratory/expiratory ratio: 0.5, and inspiratory pause time/total inspiratory time ratio: 10%. Gray zones represent the inspiratory phase. Respiratory resistance could not be detected in the first 0.4 s of the expiratory phase because high airway pressure (> 4 cmH_2_O) prevents detecting respiratory resistance

This study had some limitations. This was the first study to detect respiratory resistance during mechanical ventilation using the MostGraph-01®. We followed previously described methods to measure the respiratory resistance through an endotracheal tube [23]. There were limitations in evaluating respiratory resistance during mechanical ventilation. Respiratory resistance cannot be detected during the initial 0.4 s of the expiratory phase because the high airway pressure (> 4 cmH_2_O) prevents it (Fig. 4). This delays the increased respiratory resistance. Establishing the respiratory resistance measurement under high airway pressure requires increasing the size of the oscillation speaker.

Another limitation was that PEEP was not applied when measuring respiratory resistance using the MostGraph-01® because adding a positive expiratory pressure made it difficult to detect respiratory resistance. In normal clinical situations, PEEP is needed directly after ARMs to prevent lung collapse; therefore, a better technique is required.

We did not measure PaO_2_ to evaluate the effect of the ARMs. We could not perform blood gas analyses during the transurethral resection surgeries performed during this study. This surgery was chosen to prevent the influence of respiratory status under well-controlled anesthesia. We showed that respiratory resistance was statistically significant but could not show its clinical relevance. A new study, including clinical parameters, is needed.

## Conclusions

The ARM decreased respiratory resistance and increased lung compliance during mechanical ventilation. The ARM caused the airways to reopen. Although some difficulties were noted, measuring the FOT using the MostGraph-01® is a straightforward method to evaluate changes in respiratory resistance. We showed that respiratory resistance was statistically significant but could not show its clinical relevance. This measurement may help determine the best ARM type for patients under general anesthesia.

## Data Availability

The datasets analyzed during the current study are available from the corresponding author on reasonable request.

## Abbreviations

ARMs: Alveolar recruitment maneuvers
Cdyn: Dynamic respiratory compliance
Cst: Static pulmonary compliance
FeNO: Fractional nitric oxide concentration in exhaled breath
FOT: Forced oscillation technique
PEEP: Positive end-expiratory pressure
R5: Respiratory resistance at 5 Hz
